# Community-Based Support and Social Services and Their Association with Frailty Factors in Older People with Intellectual Disability and Affective and Anxiety Disorders: A Swedish National Population-Based Register Study

**DOI:** 10.1007/s10597-021-00909-3

**Published:** 2021-11-08

**Authors:** Nadia El Mrayyan, Christina Bökberg, Jonas Eberhard, Gerd Ahlström

**Affiliations:** 1 Department of Health Sciences, Faculty of Medicine, Lund University, PO Box 157, 22100 Lund, Sweden; 2 Division of Psychiatry, Clinical Psychosis Research Unit, Region Skane and Affiliated to Department of Clinical Sciences, Lund University, 25187 Helsingborg, Sweden

**Keywords:** Depression, Anxiety, Intellectual disability, Social services, Frailty, Longitudinal register study

## Abstract

Affective and anxiety diagnoses are common in older people with intellectual disability (ID). The aim was to describe support and social services for older people with ID and affective and/or anxiety diagnoses, also to investigate in this study group the association between support and social services and frailty factors in terms of specialist healthcare utilisation, multimorbidity, polypharmacy, level of ID and behavioural impairment. Data was selected from four population-based Swedish national registries, on 871 identified persons with affective and/or anxiety diagnoses and ID. Multivariate regression analysis was used to investigate associations between frailty factors during 2002–2012 and social services in 2012. People with multimorbidity who frequently utilised specialist healthcare were less likely to utilise residential arrangements. Those with polypharmacy were more likely utilise residential arrangements, and receive personal contact. People with moderate, severe/profound levels of ID were more likely to utilise residential arrangements and to pursue daily activities.

## Introduction

As average life span increases, a new disease panorama becomes evident, such as affective and anxiety which are more common among older people with ID (Axmon et al., 2018; Cooper et al., 2007; McCarron et al., 2013). People with ID are among the most vulnerable in society due to life-long significant limitations in reasoning, learning, problem solving, and adaptive behaviour present since birth or early childhood (American Association on Intellectual & Developmental Disability, 2020; American Psychiatric Association, 2013). Hence, the Swedish disability policy (Ministry of Social Affairs, 1999), gives people with ID legal rights based on a human rights perspective designed to offer people with disabilities equal life opportunities, conditions, and full participation in society (Ministry of Social Affairs, 1999; Tøssebro, 2016). Thus, all people with ID in Sweden are entitled to daily support and social services based on their needs assessed by a social worker and on their own wishes (SFS, 1993, 2001), based on the United Nations (UN) to normalise living standards and integrate people with ID into society (United Nations, 1948, 2006). This means that the society must be designed to meet the needs of people with ID of all ages and enable them to become fully involved in society with equal living conditions. To achieve this goal the focus needs to be on identifying barriers to full participation of this group. The government, municipalities and county councils are responsible for ensuring that people with ID are guaranteed good health, economic and social stability and the possibility of leading an independent life (The National Board of Health and Welfare, 2020). Although the former, large care institutions are closed, significant differences remain in the living conditions for people with ID and the general population (Jansson, 2006; Tøssebro et al., 2012). A follow-up study after 16 years concerning living conditions after deinstitutilisation in Sweden found that people with ID lived in more restricted social enviroments, had less leisure time and had a more limited social life compared to the general population (Umb Carlsson, 2020).

Affective and anxiety diagnoses have an impact on the physical and social life, and cause additional complex health and social needs for older people with ID (Axmon et al., 2018; Cooper et al., 2007; Hermans & Evenhuis, 2014; Hermans et al., 2013; World Health Organization, 2017). Older people with ID are more likely to have inpatient and unplanned visits in psychiatric and somatic healthcare compared to people of similar ages and sex from the general population with affective and anxiety diagnoses (Ahlström et al. 2020; El Mrayyan et al., 2020). In addition, they receive more prescription of medication with increased severity of ID and with the presence of behavioural impairment and with affective and anxiety diagnoses (Axmon et al., 2019; McGillivray & McCabe, 2004; Tsiouris, 2010). Furthermore, older people with ID and psychiatric disorders are more likely to have a broad range of comorbidities, and thus be at risk for inappropriate prescription of medication, and more extended hospitalisations (Bowring et al., 2017; O'Dwyer et al., 2016; Scheifes et al., 2016). However, much is still unknown about the influence of these factors on frailty in older people with ID and their living conditions in daily life with a diagnosis of affective and/or anxiety.

The term frailty is gaining increased international attention as the population of older adults rises globally. Frail older people are more prone to adverse health outcomes than others of the same age (Clegg et al., 2013; Hoogendijk et al., 2019; Schoufour et al., 2015; WHO, 2016). The World Health Organisation (WHO) has defined frailty as a “clinically recognizable state in which the ability of older people to cope with everyday or acute stressors is compromised by an increased vulnerability brought by age-associated declines in physiological reserve and function across multiple organ systems” (Hoogendijk et al., 2019; WHO, 2016). However, despite the increased amount of research over the past three decades, consensus on a standard measurement to identify frailty has not yet been achieved (Hoogendijk et al., 2019). Negative health outcomes as a result of ageing with lifelong disability mean a risk of more medication, increased sickness and more frequent hospitalisations. The frailty conditions develop in earlier ages in people with ID, which may lead to discrepancies in the use of healthcare compared to the general population (Evenhuis et al., 2012; Gale et al., 2015; Ouellette-Kuntz et al., 2019; Schoufour et al., 2013, 2015). The more problems present, the frailer are people with ID compared to the general population of the same age (Schoufour et al., 2013, 2014). Furthermore, WHO called attention to the fact that older people with ID are a neglected group and at risk of being treated unfairly when it comes to the provision of care and welfare (WHO, 2000).

Older people with ID have, apart from their long-standing disability, a higher risk of multimorbidity (McCarron et al., 2013), medication use (O'Dwyer et al., 2016), negative health outcomes and higher mortality than people in the general population of the same age (Ng et al., 2017). They have experienced negative psychological and environmental factors (Bond et al., 2020) throughout their lives. All this contributes to increased frailty in older age. Polypharmacy, defined as the use of five or more medications (Socialstyrelsen, 2010, 2013, 2014), is common in older people with ID and it is associated with a wide range of side-effects that increase the risk of falls and injuries (Axmon et al., 2017; Cox et al., 2010; Ham et al., 2014; Schulte & Hser, 2013).

Furthermore, in our previous research, we have investigated the health of people with ID and affective or anxiety diagnoses regarding comorbidity, healthcare utilisation and polypharmacy (Axmon et al., 2019; El Mrayyan et al., 2019, 2020). Our results confirmed previous research indicating a great amount of multimorbidity and specialist healthcare among people with ID. However, it is still unknown whether the variations in health determine the need for community-based support and social services among people with ID and affective or anxiety diagnoses. Therefore, the study aim was to describe support and social services for older people with ID and affective and/or anxiety diagnoses, also to investigate in this study group the association between support and social services and frailty factors in terms of specialist healthcare utilisation, multimorbidity, polypharmacy, level of ID and behavioural impairment.

## Methods

Population-based study on longitudinal data from four Swedish national registries.

### The Context for People with ID

People with ID in Sweden are entitled to support and social service according to the act concerning support and services for persons with certain functional impairment (abbreviated as LSS act), (SFS, 1993), and can receive complimentary services from the social services act (SoL) according to their needs (SFS, 2001). The LSS act sets out the rights for people with extensive permanent and long-standing functional impairment and disabilities for special support and social service to provide equal living conditions as others in the population. The LSS act is applied to three groups; one of these groups are persons with ID, autism, or conditions resembling autism (ASD), (Person group 1).

### Identification of the National Study Population

A national cohort of older people with ID (Person group 1) aged 55 years or older with a least one support and social service according to the LSS act at the end of 2012 were identified (N = 7936) through the national register for support and service to people with ID, abbreviated as the LSS-register. The inclusion age from 55 years is based on previous research that found premature ageing in people with ID than those without ID (Coppus, 2013). A reference group was established from the general population through one-to-one matching by year of birth and sex. In total, 7936 in the ID cohort and an equal number in the reference group were identified. The matching of people between different registries was based on the unique identification number assigned to each person living in Sweden. More details about the national cohorts are to be found in previous articles from the same project (Ahlström et al., 2020; Axmon et al., 2018). The data were delivered in 2014–2015 after ethical approval and separate secrecy reviews by the two register holders, the National Board of Health and Welfare (that identified the national cohort of people with ID and matched data from different registers to those people) and Statistics Sweden (that selected and matched the reference group of the general population, only included in previous substudies in the same project), both authorities with commissions from the government.

### Selection of the Sample for This Study

From the national population with ID (n = 7936) those with at least one affective (F3) and/or anxiety (F4) diagnosis were identified during the study period 2002–2012 through the register for specialist healthcare (National Patient Register, NPR). The diagnoses are entered in the NPR in conjunction with the utilisation of specialist healthcare, having been made by the physician with main medical responsibility for the patient. This resulted in 871 people in the ID cohort with one or both of these diagnoses in the study group. Of those, 437 (50%) were male and 434 (50%) female. The median age in the study group at the end of 2012 was 61 years (range 55–91), among those with affective disorders 61 and among those with anxiety disorders 62. The mean age at the end of 2012 for these sub-groups was 63 and 62 years respectively. Subgroups were made based on those who only have affective diagnosis (n = 400), only anxiety diagnosis (n = 293) and both affective and anxiety diagnoses (n = 178).

The matched reference group from the general population described above is not included in this sub-study, being outside the aim.

### Outcomes Variables Included in the Study From The registers

The included data were selected from four national registries based on mandatory registration.

#### LSS Registry

The seven measures included from LSS register in this study are for adult people (Table 1).

**Table 1:** Explanation of support and social services measures from the LSS and complimentary SoL-register for older people with ID

	Description of measure
LSS measures	
Personal assistance	Persons with major function impairment and extensive need for support and help in their everyday live for those who need assistance for less than 20 h a week
Companion services	Person who are not entitled to personal assistance may instead be entitled to companion service adapted to individual needs, which makes it easier to participate in the life of community
Personal contact	Help the individual to lead an independent life by reducing social isolation, helping the person participate in recreational activities, and providing advice in everyday situations (this support can be provided by a family)
Relief services	Provided regularly and during unexpected situations in the home
Short stay away from home	Can be in another family or during a stay in holiday camp
Residential arrangement with special services for adults	Services and support by staff in group accommodation. Group accommodation consists of five to eight connecting separate flats (each person one flat) in a residential area. Individuals provided service and support by staff 24 h day according to the needs of the residents. Service housing is available for individuals who are more independent. It consists of separate flat spread out in larger area and the staff can be available 24 h a day
Daily activities	This measure is entitled to people of working age who have no job or meaningful occupation and who are not participating in a training course or other type of education
SoL support and social services	
Companion service	Companion person (a home help service decision)
Daily activities	Provide assistance in employment, community, treatment, or rehabilitation outside of one's own home
Personal contact	Person or family with the task of supporting and helping individuals and their relatives in personal matters
Relief service	The release of relatives in the home (a home help service decision)
Home help service	Service concerns service in the persons home such as personal activities of daily living (ADL)
Home help service	Services concerns personal care such as cleaning
Home help service	Services concerns food distribution
Short stay away housing	Means assistance in the form of temporary housing for relief and exchange
Special housing for service and care	Municipality provide the services, special types of housing for service and care
Safety	Assistance with the security alarms
Residential services in daily living	Services for people with psychiatric, neuropsychiatric, or cognitive disability consist of support at home, support in functional social life, and support to have contact with authorities and healthcare institutions

#### SoL Registry

The SoL register includes information about support and social services provided according to the Social services act (SFS, 2001) to older people and people with disabilities who have difficulties in everyday life. Measures included (Table 1) are available from 2007 to 2012.

Both the LSS and SoL register contains information on the support and social services provided by the municipality.

#### National Patient Register

From the National patient register (NPR) was information included about inpatient and outpatient specialist healthcare, and diagnosis during 2002–2012. The NPR register is based on mandatory registration of all healthcare visits in Sweden and medical data as one primary diagnosis (main cause to the visit) and up to 21 secondary diagnoses (contributed causes to the visits) for inpatient. Outpatient healthcare consists of one primary and up to 18 secondary diagnoses. The information about diagnoses is based on International Classification Disease (ICD-10) codes. Thus, the information about healthcare visits, multimorbidity, level of ID and behavioural impairment data were collected from NPR, see Table 2.

**Table 2:** Overview of frailty factors included in the regression analysis with support and social services measures in 2012

Selected variables from registers	Description of frailty factors	Dichotomous coding of the variables
NPR register		
Specialist healthcare visits	Total sum of inpatient and outpatient healthcare visits for each person during 2002–2012	High utilisation < 12 visits, low ≥ 12 based on median
Multimorbidity	Two or more diagnoses (Kinnear et al., 2018), during 2002–2012 based on 2 digits from ICD10	< 2 and ≥ 2 diagnoses
Level of ID	At least one diagnosis of ID (F70-F79); Mild (F70), moderate, severe, and profound (F71, F72 and F73), based on at least one registration during the 2002–2012	Mild, moderate, and severe and profound
Behavioural impairment	Based on at least one registration of four digits diagnoses during the period	With and without
Prescribed drug register		
Polypharmacy	Five or more prescriptions of medication for drugs selected from an ageing perspective by an experienced geriatrician, based on at least one year with polypharmacy during the 2006–2012 (Socialstyrelsen, 2010, 2013, 2014)	< 5 and ≥ 5 prescribed drugs

#### Prescribed Drug Register

The drug prescription register contains dispensed prescribed drugs from the pharmacies. The prescribed drugs are based on the anatomical therapeutic chemical (ATC) classification and coded according to different levels of organ or system they act. The information about polypharmacy data (Table 2) was available from 2006 to 2012.

The selected drugs for this study were made by a geriatrician (SL) with experiences of treating older people with ID and engaged in a committee that decided drugs guidelines. The selected drugs of importance for older people with ID were; Alimentary tract and metabolism (A02BC, A06A, A10A, A10B, A12A), Blood and blood forming organs (B01A, B01AC, B03A, B03B), Cardiovascular system (C01AA, C01D, C03, C07, C08, C09, C10AA), Genitourinary system and sex hormones (G04BD, G04C), Systematic hormonal preparation, excluding sex hormones and insulin (H03AA), Musculoskeletal system (M01A, M05B), Nervous system including pain drugs and antipsychotics (N02A, N02B, N02C, N03A, N04B, N05A, N05B, N05C, N06A, N06BA, N06D), Respiratory system (R03), and Sensory organs (S01E).

### Statistical Analysis

Descriptive statistics included the frequencies and percentage of support and social services (LSS and SoL) in older people with ID and with affective and/or anxiety diagnoses. People with affective and/or anxiety diagnoses and received support and social services from LSS in 2012 were inserted further in a multivariate logistic regression analysis to investigate the association with the frailty variables (Table 2).

Regarding *specialist healthcare visits*, 2% were inpatient only, 24% were outpatient only, and 75% had inpatient and outpatient visits. In the analysis, inpatient and outpatient visits were merged as most of the people have both visits. In the variable *multimorbidity*, the diagnoses of affective and anxiety disorders, ID, ASD and Q90 (Down’s syndrome) were excluded (inclusion criteria for the study group). Regarding *polypharmacy* [common drugs in the general older population selected by a geriatrician (Table 2)], i.e. the number of study persons with five or more prescriptions, in this study during at least one of the years 2006‒2012. The *level of ID* was identified by the F7 (ID diagnosis): mild level (F70, n = 229) and moderate/severe/profound (F71, F72 and F73, n = 159). Among those with level of ID diagnoses, we identified those with *behaviour impairment* diagnoses (n = 133) and those without (n = 255).

The adjustment with age (was used as a continuous variable) and sex was performed in the logistic regression analysis with frailty factors and support and social service. All analyses were performed using IBM SPSS version 25. *P* values less than 0.05 were considered statistically significant.

### Ethics Approval and Consent to Participate

The study was approved by the Regional Ethical Review Board in Lund (diary no. 2013/15), and the research was performed in accordance with the Declaration of Helsinki (World Medical Association 2013). Both the National Board of Health and Welfare and Statistic Sweden made separate secrecy reviews, and the data were anonymized before being given to the PI. The study design did not include informed written consent from the participants; instead, the Ethical Review Board required that the planned study should be advertised in two national newspapers in Sweden, and that the advertisement should include information about how to withdraw from it. One of these newspapers (*Unik*) was published by the Swedish National Association for Persons with Intellectual Disability, an association for people with ID, next of kin and supporting staff within disability service. The text in this newspaper was in an easy-to-read version. The other newspaper was a major national daily (*Dagens Nyheter*). No one refused participation.

## Results

The most common support and social service from LSS for all groups of affective and/or anxiety diagnoses were residential arrangement, daily activities, and personal contact respectively during 2004–2012, and the same pattern except a lower proportion of daily activities in 2012 (Table 3).

**Table 3:** Persons with ID with at least one affective and/or anxiety diagnosis who received at least one support and social service through LSS during 2004–2012 and in 2012

Intellectual disability support according to LSS	2004–2012					Only year 2012
	Only affective diagnosis and ID (n = 400) n (%)	Only anxiety diagnosis and ID (n = 293) n (%)	Both affective and anxiety diagnoses and ID (n = 178) n (%)	Total affective and/or anxiety diagnoses and ID (n = 871) n (%)		Total affective and/or anxiety diagnoses and ID (n = 871) n (%)
Personal assistance	17 (4)	16 (5)	7 (4)	40 (5)		16 (2)
Companion service	73 (18)	65 (22)	40 (23)	178 (20)		67 (8)
Personal contact	272 (68)	190 (65)	111 (62)	573 (66)		414 (47)
Relief service	< 5	< 5	< 5	< 5		< 5
Short stay away	8 (2)	10 (3)	< 5	21 (2)		< 5
Residential arrangement	327 (82)	212 (72)	114 (64)	653 (75)		612 (70)
Daily activities	328 (82)	214 (73)	123 (69)	665 (76)		478 (55)

The results regarding complimentary support and social services from SoL are shown in Table 4. The total number of support and social services from SoL were 426 (49% of 871) people with affective and/or anxiety diagnoses for the available period 2007–2012. The social services most commonly provided were home help service, safety, residential service in daily living, and special housing in all study groups with the diagnoses of affective and/or anxiety, (Table 4).

**Table 4:** Persons with at least one affective and/or anxiety diagnosis among older people with ID who received at least one support and social service through SoL during 2007–2012 (excluding 2009) and in 2012

Support and social service according to SoL (2007–2012)	2007-2012^a^				Only year 2012
	Only affective diagnosis and ID (n = 159) n (%)	Only anxiety diagnosis and ID (n = 138) n (%)	Both affective and anxiety diagnoses and ID (n = 129) n (%)	Total affective and/or anxiety diagnoses and ID (n = 426) n (%)	Total affective and/or anxiety diagnoses and ID (n = 426) n (%)
Companion service	25 (16)	11 (8)	18 (14)	54 (13)	21 (5)
Daily activities	13 (8)	7 (5)	18 (14)	38 (9)	10 (2)
Personal contact	11 (7)	6 (4)	11 (8)	28 (7)	10 (2)
Relief service	< 5	< 5	< 5	7 (2)	5 (1)
Home help service (Personal ADL, cleaning and food distribution)^b^	92 (58)	82 (59)	65 (50)	239 (56)	131 (31)
Short stay away	13 (8)	9 (6)	10 (8)	32 (7)	14 (3)
Special housing	40 (25)	27 (20)	28 (22)	95 (22)	58 (14)
Safety	60 (38)	46 (33)	32 (25)	138 (32)	97 (23)
Residential service in daily living	46 (29)	37 (27)	56 (43)	139 (33)	102 (24)

^a^Data from 2009 were excluded due to poor quality of data

^b^We have aggregated three measures of the home help service variables to one service variable; personal activities of daily living (ADL), cleaning and food distribution

Between 80 and 90% of people with affective and/or anxiety and multimorbidity or polypharmacy, receives companion services, personal contact, residential arrangements, and daily activities (Table 5).

**Table 5:** Descriptive data about frailty factors in older people with ID, and with at least one affective and/or anxiety diagnosis who received at least one support and social service through LSS in 2012

Frailty factors		Total group with affective and/or anxiety (n = 871)									
		Personal assistance		Companion service		Personal contact		Residential arrangement		Daily activities	
		Yes (16)	No (855)	Yes (n = 67)	No (n = 804)	Yes (n = 414)	No (n = 457)	Yes (n = 612)	No (n = 259)	Yes (n = 478)	No (n = 393)
		n (%)	n (%)	n (%)	n (%)	n (%)	n (%)	n (%)	n (%)	n (%)	n (%)
Specialist healthcare visits^a^	(< 12)^d^	8 (50)	423 (50)	28 (42)	403 (50)	211 (51)	220 (48)	318 (52)	113 (44)	246 (51)	185 (47)
	(≥ 12)	8 (50)	432 (50)	39 (58)	401 (50)	203 (59)	237 (52)	294 (48)	146 (56)	232 (49)	208 (53)
Multimorbidity^b^	(< 2)^d^	< 5	67 (8)	< 5 (1)	67 (8)	30 (7)	38 (8)	55 (9)	13 (5)	46 (10)	22 (6)
	(≥ 2)	15 (94)	788 (92)	66 (99)	737 (92)	384 (93)	419 (92)	557 (91)	246 (95)	432 (90)	371 (94)
Polypharmacy^c^	(< 5)^d^	< 5	111 (13)	13 (19)	102 (13)	44 (11)	71 (15)	59 (10)	56 (22)	73 (15)	42 (11)
	(≥ 5)	12 (75)	744 (87)	54 (81)	702 (87)	370 (89)	386 (85)	553 (90)	203 (78)	405 (85)	351 (89)
Those with ID diagnosis and affective and/or anxiety (n = 388)		n = 2	n = 386	(n = 22)	(n = 366)	(n = 185)	(n = 203)	(n = 306)	(n = 82)	(n = 222)	(n = 166)
Level of ID	Mild^d^	< 5	227 (59)	16 (73)	213 (58)	116 (63)	113 (56)	164 (54)	65 (79)	119 (54)	110 (82)
	Moderate/severe/profound	< 5	159 (41)	6 (27)	153 (42)	69 (37)	90 (44)	142 (46)	17 (21)	103 (46)	56 (18)
Behavioural impairment	With^d^	< 5	133 (35)	5 (23)	128 (35)	61 (33)	72 (35)	111 (36)	22 (27)	84 (38)	49 (29)
	Without	< 5	253 (65)	17 (77)	238 (65)	124 (67)	131 (65)	195 (64)	60 (73)	138 (62)	117 (71)

^a^Cut-off for categories based on median (12 visits)

^b^Cut-off for categories based on 2 digits of ICD 10

^c^Cut-off categories based on 5 or more drugs prescription

^d^Reference group for regression analysis in Table 6

The adjusted results of the logistic regression analysis in Table 6 shows that people with more than 12 visits to specialist healthcare were less likely to live in residential arrangement compared to the ones with less than 12 visits. Also, people with multimorbidity were less likely to receive residential arrangement and daily activities compared to the ones without multimorbidity. People with affective and anxiety diagnoses and with polypharmacy were more likely to have personal contact and residential arrangement than those without polypharmacy, (Table 6). Additionally, they were less likely to receive companion service compared to those without polypharmacy.

**Table 6:** Regression analysis (OR, 95% CI) of association between frailty factors and support and social services through LSS in 2012 among older people with ID with at least one affective and/or anxiety diagnosis

Frailty factors	Total group with affective and/or anxiety (n = 871)									
	Personal assistance		Companion service		Personal contact		Residential arrangement		Daily activities	
	Unadjusted OR, 95% CI^e^	Adjusted OR, 95% CI^e^	Unadjusted OR, 95% CI^e^	Adjusted OR^d^, 95% CI^e^	Unadjusted OR, 95% CI	Adjusted OR^d^, 95% CI^e^	Unadjusted OR, 95% CI	Adjusted OR^d^, 95% CI^e^	Unadjusted OR, 95% CI	Adjusted OR^d^, 95% CI^e^
Specialist healthcare visits^a^	1.14 (0.39–3.34)	1.10 (0.37–3.27)	1.44 (0.84–2.46)	1.43 (0.83–2.47)	0.80 (0.60–1.06)	0.79 (0.59–1.05)	**0.59 (0.43**–**0.82)**	**0.63 (0.46–0.88)**	0.94 (0.71–1.25)	0.76 (0.55–1.04)
Multimorbidity^b^	N/C^f^	N/C^f^	N/C^f^	N/C^f^	1.15 (0.68–1.94)	1.14 (0.67–1.93)	**0.47 (0.24**–**0.92)**	**0.48 (0.24–0.94**)	0.61 (0.35–1.05)	**0.54 (0.30–0.99)**
Polypharmacy^c^	N/C^f^	N/C^f^	**0.45 (0.22**–**0.89)**	**0.44 (0.22–0.88**)	**1.65 (1.08**–**2.50)**	**1.63 (1.07–2.50)**	**3.57 (2.30**–**5.54)**	**3.21 (2.06–5.01)**	0.72 (0.47–1.10)	1.08 (0.68–1.70)
*Those with ID diagnosis and affective and/or anxiety (n* = *388)*										
Level of ID										
Mild	N/C^f^	N/C^f^	1.75 (0.66–4.65)	1.56 (0.57–4.23)	1.32 (0.87–2.00)	1.31 (0.86–2.00)	**0.31 (0.17–0.56)**	**0.31 (0.17–0.56)**	**0.62 (0.40**–**0.94)**	**0.60 (0.37–0.96)**
Moderate/severe/profound	N/C^f^	N/C^f^	0.57 (0.21–1.51)	0.63 (0.23–1.72)	0.75 (0.49–1.14)	0.76 (0.49–1.15)	**3.17 (1.76**–**5.71)**	**3.19 (1.75**–**5.80)**	**1.61 (1.05–2.46)**	**1.66 (1.03–2.68)**
Behavioural impairment	N/C^f^	N/C^f^	0.61 (0.21–1.72)	0.65 (0.23–1.86)	0.95 (0.61–1.46)	0.95 (0.62–1.47)	1.25 (0.71–2.19)	1.30 (0.73–2.30)	1.31 (0.89–2.05)	1.24 (0.76–2.03)

Statistically significant differences are marked in bold. The reference group is identified in Table 5

^a^Cut-off for categories based on median (12 visits)

^b^Cut-off for categories based on 2 digits of ICD 10

^c^Cut-off categories based on 5 or more drugs prescription

^d^Adjusted for age and sex

^e^Odds Ratio with 95% confidence intervals

^f^N/C (Not calculated due to small frequency less than 5)

People with moderate/severe and profound level of ID were more than three times likely to live in a residential arrangement and more than one time likely to have daily activities compared to those with mild level of ID (Table 6).

## Discussion

To the best of our knowledge there are no previous studies investigating the association between frailty factors and support and social services for older people with ID and with affective and/or anxiety diagnoses. The main findings of this study show that older people with ID and affective and/or anxiety diagnoses and with polypharmacy were more likely to live in residential arrangement and receive personal contact compared to those without polypharmacy. In addition, those with multimorbidity and high specialist healthcare visits were less likely to live in residential arrangement compared to those without multimorbidity and lower specialist healthcare visits. Furthermore, those with multimorbidity were less likely to receive daily activities compared to those without multimorbidity. Moreover, our results showed that those in the study group with moderate/severe/profound level of ID were more likely to live in residential arrangement and get daily activities compared to those with mild level of ID.

Our results about frailty factors imply high prevalence of polypharmacy, which is in line with previous studies stating that, polypharmacy, especially psychotropic drugs are prevalent and high in people with ID (Häbler et al., 2015; Hsu et al., 2014; O'Dwyer et al., 2016; Sheehan et al., 2017; Tsiouris et al., 2013). This has been explained by, high prevalence of psychiatric diagnoses, the presence of behavioural impairment and with severe level of ID (Bowring, et al., 2017; O'Dwyer, et al., 2016; Sheehan et al., 2015; Tsiouris, et al., 2013). Vetrano et al. (2013) found in seven European countries excessive prescription of polypharmacy after hospitalisation during the past 90 days in older people with cognitive impairment in residential arrangements. Hospitalisation could also explain our results of more likely having polypharmacy when they live in residential arrangements, as when they are discharged from hospitals, they will receive medication prescription to better control their symptoms. Moreover, probably because the compliance of medication is higher for people with ID when staff are responsible for pick up the prescribed medication of healthcare from the pharmacy and distribute these to the person with ID and with affective and anxiety diagnoses.

The high prescription of psychotropic medication reflects the increase in psychiatric disorders among older people with ID. In spite of the lack of their limited effectiveness, this can lead to several side effects such as decreased interest or lethargy, reduced mobility, more isolation and changed perception of surrounding and relationship with others in older people with ID (Scheifes et al., 2016; Tsiouris, 2010). Thus, because of these side effects, older people with ID might be less engaged in daily activities and their needs will not be met by staff working in residential arrangements (Bond et al., 2020; Innes et al., 2012). This is in line with our results that those in the study group with polypharmacy are more likely to receive personal contact services to acquire assistance in participating in activities compared to those without polypharmacy.

The high prevalence of multimorbidity for those who live in residential arrangement rises a concern about negative health outcomes in older people with ID. The high prevalence of multimorbidity (Cooper et al., 2015; Hermans & Evenhuis, 2014; Kinnear et al., 2018; McCarron et al., 2013) make the identifying of psychiatric diagnoses very difficult in people with ID, due to masked and overshadowing symptoms with behavioural impairments and communication problems (Bond et al., 2019; Mason & Scior, 2004; O'Dwyer et al., 2018). Therefore, due to these difficulties their ability to describe their symptoms is dependent on how well the staff working in residential arrangements can identifying needs. This may lead to a more severe stage of disease progression, which is expected to be associated with more visits to specialist healthcare. This could explain our findings that the high prevalence of multimorbidity increases the risk to specialist healthcare visits to receive treatment for their problems and may explain less likely to live in a residential arrangement.

Additionally, we have previously found that older people with ID with affective and anxiety diagnoses were more likely to have inpatient and unplanned healthcare visits in specialist healthcare (El Mrayyan et al., 2020). The unplanned specialist healthcare visits could be indicator of difficulties that staff in residential arrangement face and refer them to specialist healthcare due to unfulfilled needs. In the presence of multimorbidity, the persons with ID experience more complex problems and it is therefore more challenging to meet their needs. This can explain our results that those with multimorbidity are less likely to receive daily activities compared to those without multimorbidity. Our study revealed that those with moderate/severe/profound level of ID and with behavioural impairment are more likely to live in residential arrangement and this could mean that the staff have experiences of identifying the behavioural impairments. This could indicate that the staff in residential arrangement were not able to identify their health problems at an early stage, and instead described these as behavioural problems and referred these persons for further treatment in specialist healthcare.

In addition, we found that, about 75% of the study group were living in residential arrangements and receive daily activities. This result is in line with reports from the National Board of Health and Welfare in Sweden which reported that the most common support and social services provided by LSS were assistance in residential arrangements with special services and daily activities for adults with ID (Socialstyrelsen, 2019). In contrast, the results of this study showed that the less common support and social services were personal assistance and companion service for those with affective and/or anxiety diagnoses. Furthermore, those with polypharmacy were more likely to receive personal contact than those without polypharmacy. This indicates that the 24-h support of staff from the residential arrangements are not enough for the needs people with affective and/or anxiety diagnoses they might have. Also, might be that those living in residential arrangements around 54% have mild level ID and thus able to reflect on their situation and realise their difficulties. A recent study found that most of older people with ID and with depression and/or anxiety symptoms have inactive lifestyle, troubles eating meals or engaging in social activities and difficulties in carrying out the activities of daily living (ADL) and need more support from staff working in community or home care compared with those without these diseases (Bond et al., 2020). Moreover, those depressed or with anxiety who lives in their homes, would need support from persons they confide in such as a family, or get to known staff working in community (McCausland et al., 2016). Their increased needs of support indicates by our results that the most common support and social services were home help services, residential service in daily living, safety and special housing from SoL as a complementary service.

Staff in residential arrangements play a crucial role in maintaining the wellbeing of older people with ID (Kåhlin et al., 2016; Tøssebro et al., 2012; Webber et al., 2014, 2016). For example, if a person with ID has communication problems and cannot show feelings and desires with the body, or by facial expression, the person needs a lot of assistance from staff who knows him well. At residential arrangement, the staff who works regularly know the residents well and are experienced enough to read the person’s facial and body signs for communications. However, the staff is not equally accustomed to identifying symptoms of emerging health problems. In Sweden, as in many other countries, there are no specific requirements of specialised education for staff in residential arrangements who work with people with complex needs such as people with ID (Emilsson, 2009; Martin et al., 2018; Northway et al., 2016). Hence, the general education for the social care workers will not provide staff with the specific knowledge and skills they need to handle the complex health needs in people with ID. This could be incompatible with the important role the staff play in monitoring the health status of people with ID and in identifying early symptoms or problems to improve the quality of life for this frail population. Several studies reported challenges such as lack of knowledge, skills, misunderstanding and miscommunication which leads to lack of confidence in care delivery among people with ID in residential arrangements (Ann & Helena, 2014; Costello et al., 2007; Innes et al., 2012; Kåhlin et al., 2016; Quigley et al., 2001). This highlights the importance of training the residential social care workers in terms of frailty and health issues of people with ID (Northway et al., 2016). Increase knowledge and skills could reduce the health inequalities people with ID, develop strategies with meaningful care planning according to their needs to improve their wellbeing and quality of life (Emerson & Baines, 2011). Bedsides necessary knowledge and skills of support and social services in residential arrangements, the social care workers need knowledge of existing resources in the healthcare system (Alftberg et al., 2019; Holst et al., 2018). This type of expertise promotes greater integration and collaboration with other professional and being a part of the team to meet the needs of older people with ID and with complex, comorbid and challenging presentation and deliver the best quality of care (Alftberg et al., 2019; Holst et al., 2018; Northway et al., 2016).

### Methodological Consideration

The potential strength of the study is that it included data from mandatory national registries with high validity and population coverage in Sweden (Ludvigsson et al., 2016). However, the use of the LSS register as a proxy for ID is assessed as more valid than in the NPR registry when ID diagnosis is only included as a main or contributed reasons to visiting specialist healthcare. To identify and register comorbidity associated with ID requires a high level of specialist expertise, which most physicians today miss due to the lack of specialist training. This can explain the low amount of registrations of ID as causes to the healthcare visit.

There are further limitations that readers must consider. The personal assistance for more than 20 h a week is not included in the LSS register since the data is only available in other registers by the social security agency. However, a low proportion of personal assistance is expected as most older people with ID live in residential arrangement (75%) and have staff available 24 h; thus, they are not entitled to personal assistance. Most older people with ID who grew up and lived in institutions had moved already to residential arrangements in the society before personal assistance became an alternative LSS service for people living in own home (Clevnert & Johansson, 2007).

Another limitation was the frailty factors identification. This study includes data of only specialist healthcare (inpatient and outpatient) visits, and information about primary healthcare was not available at a national level, and the association between support and social services and primary healthcare is therefore not included. Hence, most people in the general population with affective and anxiety diagnoses are examined or followed up in primary healthcare (Sundquist et al., 2017). However, older people with ID are a frail group with more complex needs and therefore expected to have more visits to specialist healthcare. Also, in a previous study concerning this study group it was found that a high proportion (some 70%) utilised specialist healthcare during the eleven-year study period (El Mrayyan et al., 2020).

Finally, this study included selected specific drugs that are of importance for ageing people and those with ID. Even if some drugs therefore can be underestimated, we assess it not as a threat for the validity of the study due to agreement results with the previous literature. The frailty factors were based on data from 2002 to 2012, except polypharmacy which cover a shorter period 2006–2012 due to the availability of the data in the prescribed drug register. Regarding the different study period for polypharmacy, we think that it will not affect the validity since it is likely that most persons have it also before 2006. Drug reviews of treatment are not regularly made for persons utilising residential arrangements, which means that it can go on for many years if nothing special happens that gives a reason for such review.

## Conclusion

The findings confirmed that people with ID and with affective and anxiety diagnoses living in residential arrangements have several frailty factors that indicate complex needs of support and social service. Identifying and understanding the consequences of frailty constitutes the necessary basis for developing strategies and intervention to support them and reduce the risk of adverse outcomes. The staff working in residential arrangement need to have high competences and knowledge of frailty in each unique person to be able to meet complex needs to improve their health and quality of life.
